# Improved Patient-Reported Medication Adherence, Patient Satisfaction, and Glycemic Control in a Collaborative Care Pharmacist-Led Diabetes “Tune-Up” Clinic

**DOI:** 10.3390/ijerph18179242

**Published:** 2021-09-01

**Authors:** Jan D. Hirsch, Nancy Kong, Kevin T. Nguyen, Christine L. Cadiz, Crystal Zhou, Sarah A. Bajorek, Mark Bounthavong, Candis M. Morello

**Affiliations:** 1 Department of Clinical Pharmacy Practice, University of California Irvine School of Pharmacy and Pharmaceutical Sciences, Irvine, CA 92612, USA; christine.cadiz@uci.edu; 2 Department of Pharmacy, San Francisco Veterans Affairs Health Care System, San Francisco, CA 94121, USA; Nancy.Kong@va.gov; 3 Department of Pharmacy, University of California San Diego Health, San Diego, CA 92103, USA; ktn001@health.ucsd.edu; 4 Department of Clinical Pharmacy, University of California at San Francisco School of Pharmacy, San Francisco, CA 94143, USA; crystal.zhou@ucsf.edu; 5 Department of Pharmacy, University of California Davis Health, Sacramento, CA 95817, USA; sabajorek@ucdavis.edu; 6 Pharmacy Benefits Management Academic Detailing Service, U.S. Department of Veterans Affairs, San Diego, CA 92161, USA; mbounthavong@health.ucsd.edu; 7 Division of Clinical Pharmacy, University of California San Diego Skaggs School of Pharmacy and Pharmaceutical Sciences, La Jolla, CA 92093, USA; cmmorello@health.ucsd.edu; 8 Department of Pharmacy, Veterans Affairs San Diego Healthcare System, San Diego, CA 92161, USA

**Keywords:** medication adherence, diabetes, pharmacist–patient relations, patient satisfaction, pharmacist, glycemic control, collaborative care

## Abstract

Diabetes complications remain a leading cause of death, which may be due to poor glycemic control resulting from medication nonadherence. The relationship between adherence status and HbA1c (glycemic control) has not been well-studied for clinical pharmacist interventions. This study evaluated medication adherence, patient satisfaction, and HbA1c, in a collaborative pharmacist-endocrinologist diabetes clinic over 6 months. Of 127 referred, 83 patients met the inclusion criteria. Mean medication adherence scores, considered “good” at baseline, 1.4 ± 1.2, improved by 0.05 points (*p* = 0.018), and there was a 26% increase in patients with good adherence. A significant improvement of 0.40 percentage points (95% CI: −0.47, −0.34) was observed in mean HbA1c across the three time points (*p* < 0.001). Mean total satisfaction scores were high and increased, with mean 91.3 ± 12.2 at baseline, 94.7 ± 9.6 at 3 months, and 95.7 ± 10.8 at 6 months (*p* = 0.009). A multimodal personalized treatment approach from a pharmacist provider significantly and positively impacted glycemic control regardless of self-reported medication adherence, and patient satisfaction remained high despite changing to a clinical pharmacist provider and increased care intensity.

## 1. Introduction

Diabetes is a chronic disease associated with many comorbidities, which all may require intensive treatment approaches involving lifestyle changes and complex medication regimens. According to the Centers for Disease Control, there are currently 34.2 million people that have diabetes and 88 million that have prediabetes living in the United States (US). Despite the continuous development of new diabetes drugs, diabetes still remains the 7th leading cause of death in the US, which may be due to a lack of glycemic control [1]. It is estimated that over 45% of patients with type 2 diabetes fail to ever achieve glycemic control, which may be attributed in large part to poor medication adherence [2]. As individual patient medication regimens become more complex, the risk of potential non-adherence can increase [3]. Studies have indicated that medication nonadherence serves as a significant barrier to achieving glycemic control [2,3,4]. Egede and colleagues performed a longitudinal retrospective cohort study at a southeastern Veterans Affairs (VA) facility, where they analyzed the Medication Possession Ratio (MPR) in adult patients with type 2 diabetes, with lower MPR representing poorer adherence. These researchers found that every percentage increase in the MPR conferred 48% lower odds for poor glycemic control [5]. Glycemic control worsened over time if medication nonadherence was present. 

Pharmacists are well-positioned to help manage diabetes and address education and medication adherence-related issues. Two meta-analyses related to pharmacist-provided interventions reported positive results. One found that pharmacist-led diabetes education regarding disease complications, medication adherence, lifestyle modifications, and education about self-management skills, led to a mean HbA1c reduction of 0.75%, while the other found that pharmacist interventions such as education and medication management reported a mean HbA1c reduction of 1% [6,7]. Another meta-analysis of pharmacist-led diabetes self-management education found a positive effect on HbA1c and suggested improved medication adherence [8]. Pharmacist-led self-management interventions have been reported to be three times more effective than those of other providers, such as physicians, nurses, or diabetes educators [8,9]. Interventions by pharmacists have also been shown to improve medication adherence, which can improve a patient’s overall control of their diabetes and associated comorbidities [10,11,12,13,14,15]. However, there is a lack of research in the literature studying the relationship between medication adherence and HbA1c within a pharmacist-led diabetes clinic.

The aim of this study was to evaluate self-reported medication adherence, patient satisfaction, and HbA1c over time, as well as explore the relationship between the two patient-reported outcomes and HbA1c, in patients treated in a collaborative care pharmacist-endocrinologist diabetes clinic over a period of 6 months. Our main hypothesis was that medication adherence and HbA1c would improve, while patient satisfaction with the pharmacist provider would be similar to patient satisfaction with their referring PCP. We also hypothesized that improved HbA1c would be associated with greater medication adherence and patient satisfaction. 

## 2. Materials and Methods

### 2.1. Objectives

The primary objective was to compare self-reported medication adherence, patient satisfaction, and HbA1c over time. The secondary objective was to explore the relationship between self-reported medication adherence and patient satisfaction with change in HbA1c.

### 2.2. Setting, Design, and Sample Size

This was a quasi-experimental pre–post design study that included demographic, clinical, and patient-reported data collected from chart reviews and questionnaires for patients referred to the Diabetes Intense Medical Management (DIMM) clinic at the Veterans Affairs San Diego Healthcare System (VASDHS) by their PCP within the study period. 

At the VASDHS, a collaborative physician-pharmacist (DIMM) clinic uses a “tune-up” model, with the goal of helping patients achieve metabolic control and also a focus on a holistic approach, including personalized comprehensive medication management, identifying healthy lifestyle practices, and addressing medication barriers [16]. Patients with type 2 diabetes are referred to this half-day per week clinic by their primary care provider (PCP). Overseen by an endocrinologist, the clinic has an annual capacity of about 60 patients, and offers 60 min visits at 2- to 3-month intervals. At each visit, patients are seen by a pharmacist to create individualized care plans to empower them as drivers of their health and help them achieve their metabolic goals. Once goals are achieved, patients are referred back to their PCP. Improvement in HbA1c for DIMM clinic patients has previously been shown to be greater than for a comparator group of patients attending PCP clinics after six months (−2.4 (SD = 2.1) vs. −0.8 (SD = 1.7); *p* < 0.001) [16]. The focus of this current study is on patient-reported outcomes of adherence and satisfaction that were collected specifically for the DIMM clinic as a quality improvement measure, and therefore a comparator group is not possible for this study.

Patients were included in this study if they had a diagnosis of type 2 diabetes, a HbA1c of greater than 8% at the time of referral, and were 18 years of age or older. Patients were excluded if they had HbA1c of 8% or less or did not have all adherence, patient satisfaction, and HbA1c data available for the baseline visit. Patients who were lost to follow-up at both the 3- and 6-month time points after the initial visit were not included in the final analysis. Post hoc analysis using the Wilcoxon signed rank test indicated that the total sample of 83 allowed for at least 80% power to detect a difference of 0.05 with a two-tailed test, alpha of 0.05, based on the pre-period and post-period values for the primary objective outcomes (adherence, satisfaction, and HbA1c).

### 2.3. Methods for Data Collection and Distribution

Data were collected retrospectively via paper and electronic chart review for each visit between June 2009 and November 2014. For the purposes of this study, data from patient visits at their initial visit (0 month), 3-month visit, and 6-month visit were assessed. This study was approved by the VASDHS Institutional Review Board (IRB) for the study of human subjects.

Each patient was seen at baseline when first enrolled in the clinic and at approximately 3-month intervals thereafter. Labs, including HbA1c, were obtained with each visit. At the end of each visit, as part of routine DIMM clinic operations, patients were given a two-page paper questionnaire that included the four-item Morisky Medication Adherence Scale (MMAS-4) questions and the Patient Satisfaction with Pharmacist Services Questionnaire (PSPSQ 2.0) [17,18,19]. The self-reported measure of adherence (MMAS-4) was selected because it was easy to administer in a busy clinic setting as part of routine patient care, and it captured the patient’s overall assessment of their adherence to all of their medications, as opposed to more labor-intensive tablet counting or prescription refill tracking (which for most chronic medications is automatic at VASDHS) across multiple medications. Both surveys have been previously validated in the literature and permission was obtained to use each instrument from the developers. 

The MMAS-4 is a four-item questionnaire used to assess self-reported medication adherence. The questions are phrased such that an answer of “yes” signifies nonadherence and an answer of “no” signifies adherence. A total score was calculated by totaling the number of yes answers to items on the questionnaire, with lower scores corresponding to more optimal adherence behavior and higher scores corresponding to poorer medication adherence behavior [17,18]. Similar to a previous study utilizing the MMAS-4 to assess medication adherence in patients with type 2 diabetes [20], patients were divided into two groups for the analysis: those with good adherence (scores of 0 or 1) and those with poor adherence (scores of 2 to 4). Patients were also divided into the following groups for analysis: those with improved adherence and those with no improvement in adherence from baseline. Improvement in adherence was defined as a negative change (decrease) of ≥1 in adherence score at 3 or 6 months in comparison to baseline.

The PSPSQ 2.0 is a 22-item questionnaire used to assess patient satisfaction in 4 domains: quality of care (10 items), interpersonal relationship with the healthcare provider (6 items), overall satisfaction (4 items), and total satisfaction (all 22 items) [19]. For each item of the PSPSQ, the patient chose from four responses: “Strongly Agree”, “Agree”, “Disagree”, and “Strongly Disagree”. Patients’ responses in each domain were then converted to a scale of 0 to 100, with 100 being the highest possible score for satisfaction. At baseline, the PSPSQ measured the patient’s satisfaction with the referring PCP. At the 3- and 6-month timepoints, the PSPSQ measured the patient’s satisfaction with the pharmacist provider in the DIMM clinic.

The last observation carried forward (LOCF) approach was used to impute missing values for questionnaire data and clinical data for patients who were unable to attend the clinic at either the 3-month or the 6-month timepoints or did not complete the questionnaire at the follow-up visit.

### 2.4. Data Analysis

Skewness testing indicated non-normal distribution for most variables and statistical tests for non-parametric data were used for analysis. Results were virtually the same but just slightly more conservative in some cases compared to parametric testing. Therefore, where applicable, significance results presented are from non-parametric tests, but means and standard deviations are presented in this paper for ease of interpretation. Chi-square and Wilcoxon Rank Sum tests were used to compare baseline characteristics between groups of patients. Generalized estimating equation (GEE) models using the linear form with autoregressive correlation were constructed to evaluate the change in outcomes (adherence, satisfaction (total and sub-domains), HbA1c) across baseline, 3, and 6 months after intervention. Clustered robust standard errors were estimated to account for the repeated measures for each patient in the panel, and results were presented as the average change with 95% confidence intervals (CI). To examine the relationship between medication adherence status and HbA1c, the Wilcoxon Rank Sum test was used to compare change in HbA1c from baseline (at 3 and 6 months) between groups of patients who had good adherence vs. poor adherence, and between groups of patients who had improvement in adherence vs. no improvement in adherence. Spearman Rank Order Correlation was used to examine the correlation between median change in HbA1c and median change in total satisfaction from baseline at 3 and 6 months. Correlations of 0.29 or less were considered to be small, those between 0.30 and 0.49 were considered moderate, and those greater than or equal to 0.5 were considered large [21]. Statistical analyses were conducted using STATA, 16.0 (College Station, TX, USA). Significance levels were set a priori at 0.05 for initial comparisons and at 0.01 for post hoc testing.

### 2.5. Participants and Recruitment

Participants were patients with type 2 diabetes referred to the DIMM clinic by their PCP, and who met inclusion criteria. Data were collected retrospectively from paper charts and electronic health records containing clinical measures and questionnaire information routinely obtained as part of patients’ medical care visits in the DIMM clinic at the VASDHS. 

## 3. Results

### 3.1. Participants

A total of 127 patients were referred to the DIMM clinic during the study period of June 2009 and November 2014. These patients completed a total of 312 surveys during their initial or follow-up visits. Of the 127 patients, 44 patients were excluded from the final analysis because they did not meet the inclusion criteria, did not have adherence, patient satisfaction, and HbA1c data for baseline, or were lost to follow-up at both subsequent time points. A total of 83 patients were included in the final analysis.

Baseline characteristics are summarized in Table 1. The majority of patients were White males, with average age of 59.8 years, and an average BMI of 33.3. Average age-adjusted Charlson Comorbidity Index (CCI) was 4.9, with more than half of the study population having at least one of the following comorbidities: hyperlipidemia, hypertension, mental illness, obesity, or neuropathy. 

### 3.2. Medication Adherence, HbA1c, and Patient Satisfaction 

At baseline, the mean (±SD) medication adherence score of 1.4 (±1.2) indicated good adherence, and in the GEE model, there was a significant decrease (improvement) in mean adherence score of 0.05 points (95% CI: −0.10, −0.01) across the three time periods (*p* = 0.018). Additionally, we reported a significant decrease (improvement) of 0.40 percentage points (95% CI: −0.47, −0.34) in mean HbA1c across the three time points (*p* < 0.001). There was a significant increase in mean total satisfaction score of 0.73 points (95% CI: +0.18, +1.28) across time points (*p* = 0.009). Similarly, mean patient satisfaction scores as represented by the sub-domains of quality of care, interpersonal relationships, and overall satisfaction significantly increased by 0.85 points (95% CI: +0.27, +1.43; *p* = 0.004), 0.61 points (95% CI: +0.05, +1.17; *p* = 0.032), and 0.72 points (95% CI: +0.09, +1.35; *p* = 0.024) respectively, across the study time periods (Table 2).

### 3.3. Medication Adherence Status

**Figure 1:**
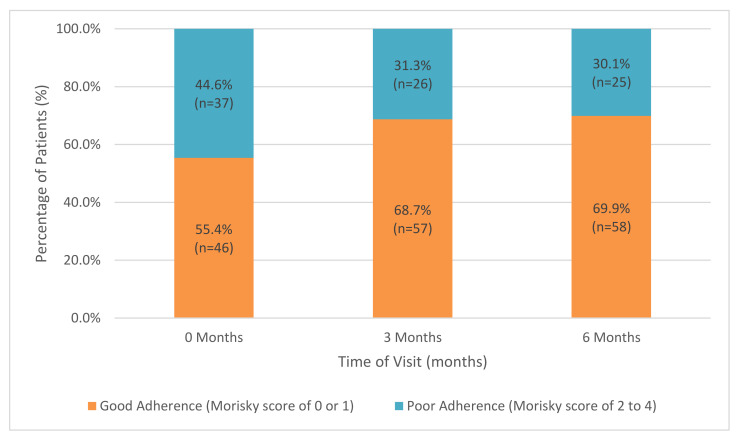
Proportion of patients with good adherence vs. poor adherence over time (n = 83).

At baseline, 55.4% of patients were categorized as having good adherence and 44.6% of patients were categorized as having poor adherence. There were no significant differences in baseline characteristics between the two groups, with the exception of BMI, which was higher in the group with poor adherence (*p* < 0.05). At 3 months, the percent of patients with good adherence increased to 68.7% and those with poor adherence decreased to 31.3%. At 6 months, the number of good adherers remained relatively unchanged, with 69.9% of patients being classified as good adherers and 30.1% of patients being poor adherers (Figure 1). From baseline to 3 months, 31.3% of patients had an improvement in adherence score and 68.7% of patients showed no improvement in adherence score. The percentages remained the same when considering improvement in adherence scores from baseline to 6 months.

### 3.4. Medication Adherence and HbA1c

Mean change in HbA1c from baseline was not significantly different between patients classified as having good adherence and patients with poor adherence at either the 3- or 6-month time points (Table 3). A mean HbA1c change from baseline to 3 months of −2.1% (±2.0) was observed in the good adherence group and −1.2% (±1.6) in the poor adherence group (*p* = 0.094). Mean change in HbA1c from baseline to 6 months was −2.6% (±2.1) in the good adherence group and −2.0% (±1.3) in the poor adherence group (*p* = 0.255). However, there was a significant difference in mean change in HbA1c from baseline to 3 months when comparing patients with improvement in adherence to those without improvement in adherence. From baseline to 3 months, a larger mean HbA1c change of −2.8% (±2.1) was observed in patients with improvement in adherence compared to the smaller mean change of −1.3% (±1.6) in patients with no improvement in adherence (*p* = 0.002). At 6 months, the mean HbA1c change from baseline in patients with improvement in adherence (−2.9% (±1.7)) was not significantly different than that observed in patients with no improvement in adherence (−2.2% (±1.9)) (*p* = 0.142) (Table 3).

### 3.5. Patient Satisfaction and HbA1c

A small negative correlation was found between mean change in HbA1c (−1.8% (±1.9)) and mean change in total patient satisfaction (+3.4 (±11.8)) from baseline to 3 months (r = −0.289, *p* = 0.008) (Table 4). No significant correlation was found between the mean change in HbA1c (−2.4% (±1.9)) and the mean change in patient satisfaction (+4.4 (±15.4)) at 6 months (r = −0.062, *p* = 0.578). 

## 4. Discussion

This study demonstrated that complex patients with type 2 diabetes, who also had a large number of medical and mental health comorbidities and were treated in a pharmacist-endocrinologist collaborative diabetes “tune-up” clinic, were able to achieve significant HbA1c reductions, irrespective of self-reported medication adherence status. All patients achieved improved glycemic control with a mean HbA1c reduction of 2.4% within a 6-month time period. Overall, mean self-reported adherence was good at baseline and did not change appreciably over time. However, we observed a 26% increase in the number of patients classified as having good adherence vs. poor adherence by the study end. 

Baseline patient satisfaction with their referring PCP was greater than 90% in all domains and remained so despite the change in provider to a clinical pharmacist and increased intensity of care in the DIMM clinic. Overall, our main hypothesis that medication adherence and HbA1c would improve, and satisfaction with the pharmacist provider would be similar to patient satisfaction with their referring PCP, was supported. Our exploratory hypothesis that improved HbA1c would be associated with greater medication adherence and patient satisfaction was partially supported in that at three months, we observed a larger mean HbA1c change for patients with improvement in adherence vs. those with no improvement in adherence, and a small correlation between increased satisfaction and improved HbA1c.

The result whereby patients who showed an improvement in self-reported medication adherence were able to achieve larger HbA1c reductions in a shorter period of time (3 months) compared to patients with no improvement in self-reported adherence warrants further investigation in larger studies, since this was an exploratory objective. Regardless, all patients (i.e., those with and without improved adherence) were able to achieve a similar HbA1c reduction (over 2%) by 6 months, which may imply that patients with no improvement in adherence eventually “caught up” in terms of HbA1c reduction. If so, improved HbA1c control seen in this study, and possibly other pharmacist clinics, may be attributed to factors aside from medication adherence alone. The success in HbA1c reduction seen in this study is likely due to a combination of many variables since the DIMM clinic pharmacist provider utilizes a multipronged personalized clinical treatment approach, including but not limited to, 60 min visits, comprehensive medication management, disease education, and assistance with implementation of lifestyle changes and medication barriers.

The findings of our study are similar to studies in the current literature showing that pharmacist-led interventions, of varying models, improved HbA1c and adherence in patients with diabetes. Meta-analyses have reported that pharmacist-led comprehensive education interventions achieve a mean HbA1c reduction of <1%, while interventions combining both education and comprehensive medication management yield an HbA1c reduction of 1%, and also improve patient-reported medication adherence where adherence was evaluated [6,7,10,11,12,13,14,15]. In the DIMM clinic, which employs both patient-specific education and comprehensive medication management, we observed a more robust mean HbA1c reduction for our patient population. In a study with a patient care intervention similar to the DIMM clinic, the authors did examine both HbA1c and self-reported adherence, although the researchers did not include exploration of the relationship between HbA1c and adherence as in our study [22]. In that study, pharmacist collaboration with physicians in the development of diabetes care plans with respect to medication management and patient education resulted in a mean HbA1c reduction of 1.7% and a significant improvement in mean self-reported adherence over a period of 6 months [22]. In comparison, in the DIMM clinic, we observed a slightly higher mean HbA1c reduction of at least 2.2% and no significant difference in mean self-reported adherence over a similar time period. Although we did not observe an appreciable improvement in mean self-reported medication adherence, we noted that the adherence level was good at baseline, and approximately one-third of our study population experienced improvement in medication adherence compared to baseline. We did not find any studies of pharmacist-led interventions examining the relationship between self-reported medication adherence and HbA1c. The high level of patient satisfaction reported with both their referring PCP and the DIMM clinic pharmacist is consistent with the high level of satisfaction reported in a medical home model that included a pharmacist within the Veteran Affairs health system. Although the study used a different satisfaction scale, mean scores were in the 90% range [23].

Limitations to be considered in our study include the non-randomized, retrospective nature of our study design that did not include a control group, and the small sample size in a single clinic with primarily White male patients within the VA health system. It was not possible to have a control group since self-reported adherence and satisfaction were only measured in the DIMM clinic as part of the normal routine clinic operations. These factors limit the generalizability of our study outcomes. Additionally, medication adherence was self-reported by the patient and not by a more stringent direct measurement, such as medication counts. Further, missing questionnaire and HbA1c data may be a study limitation affecting the representativeness of the group results, since less satisfied or adherent patients may have chosen not to complete questionnaires. Last observation carried forward was used to mitigate this issue. Moreover, since our study duration was 6 months, we did not assess long-term changes in adherence, satisfaction, or HbA1c. Future studies involving a larger study population with a comparator group and longer follow-up periods should be conducted to further elucidate the long-term impact the DIMM clinic model, or similar comprehensive pharmacist provider interventions, have on adherence and glycemic control. Additionally, the relationship between self-reported adherence and glycemic control in a larger study should also consider the possibility of a temporal factor affecting the relationship between adherence and clinical outcomes, and the influence of other factors such as comorbidities, gender, and total medication regimen complexity. Lastly, this study was performed in a veteran population at VA, which is the largest, integrated healthcare system in the US. Although generalizability may be limited to other smaller healthcare systems, large, integrated healthcare systems similar to VA may find these results useful for their population. 

## 5. Conclusions

Results from this study highlight the positive impact pharmacists can have on improving medication adherence, glycemic control, and patient satisfaction. Regardless of self-reported medication adherence status, complex patients with type 2 diabetes were able to achieve, on average, at least a 2% reduction in HbA1c within 6 months, implying that patients benefit from the optimization of medication therapy and the personalized treatment approach of the DIMM clinic. Patient satisfaction remained high despite the change in provider to a clinical pharmacist and increased intensity of care in the DIMM clinic.

## Figures and Tables

**Table 1:** Baseline characteristics of all patients included in analysis (n = 83).

Characteristic	Mean ± SD
Age (years)	59.8 ± 8.5
BMI (kg/m^2^)	33.3 ± 6.6
CCI (Charlson Comorbidity Index)	4.9 ± 2.5
** Gender **	**n (%)**
Male	82 (98.8%)
Female	1 (1.2%)
** Race **	**n (%)**
White	49 (59.0%)
African American	13 (15.7%)
Asian	8 (9.6%)
American Indian	3 (3.6%)
Native Hawaiian/Pacific Islander	3 (3.6%)
Unspecified	7 (8.4%)
** Comorbidities (Non-Diabetes) **	**% Frequency**
Hyperlipidemia	90.4%
Hypertension	88.0%
Mental Illness ^1^	74.7%
Obesity	65.6%
Neuropathy	51.8%
Coronary Artery Disease	31.3%
Retinopathy	24.1%

^1^ Includes depression, anxiety, bipolar disorder, schizophrenia, and post-traumatic stress disorder.

**Table 2:** Mean medication adherence, HbA1c%, and patient satisfaction at baseline, 3 months, and 6 months (n = 83), mean ± SD.

	Baseline (mean ± SD)	3 Months (mean ± SD)	6 Months (mean ± SD)	Average Change (95% CI) ^3^	*p*-Value ^3^
Medication Adherence Score ^1^	1.4 ± 1.2	1.1 ± 1.2	1.1 ± 1.2	−0.05 (−0.10, −0.01)	0.018
HbA1c (%)	10.4 ± 1.5	8.6 ± 1.7	7.9 ± 1.6	−0.40 (−0.47, −0.34)	<0.001
Total Satisfaction Score ^2^	91.3 ± 12.2	94.7 ± 9.6	95.7 ± 10.8	+0.73 (+0.18, +1.28)	0.009
Quality of Care	90.7 ± 12.6	94.6 ± 10.0	95.8 ± 11.2	+0.85 (+0.27, +1.43)	0.004
Interpersonal Relationship	92.9 ± 12.2	95.9 ± 9.0	96.5 ± 10.7	+0.61 (+0.05, +1.17)	0.032
Overall Satisfaction	90.9 ± 14.5	94.9 ± 8.9	95.2 ± 10.8	+0.72 (+0.09, +1.35)	0.024

^1^ Medication adherence score was calculated from the four questions of the Morisky Medication Adherence Scale (MMAS-4), a measure of self-reported medication adherence. Lower scores correspond to better medication adherence.: *Permission to use the MMAS-4 was obtained from* *Donald E. Morisky, ScD, ScM, MSPH, Professor, Department of Community Health Sciences, UCLA School of Public Health, 650 Charles E. Young Drive South, Los Angeles, CA 90095-1772*. COPYRIGHT—Morisky Medication Adherence Scale (©MMAS-4 Item). ^2^ Total Satisfaction Score is a composite of all items in the 22-item PSPSQ questionnaire, with specific questions classified within subcategories of quality of care satisfaction, interpersonal relationship satisfaction, and overall satisfaction with the provider. At baseline, patients were asked to rate satisfaction with the patient’s prior provider (PCP). At 3 and 6 months, scores reflect satisfaction ratings with the DIMM clinic provider. ^3^ Results are from generalized estimating equation (GEE) models.

**Table 3:** Mean HbA1c change over time by adherence status, mean ± SD (n = 83).

Adherence Status	HbA1C Δ from Baseline to 3 Months	*p*-Value ^1^	HbA1c Δ from Baseline to 6 Months	*p*-Value ^1^
**Good Adherence**	−2.1% ± 2.0% (n = 57)	0.094	−2.6% ± 2.1% (n = 58)	0.255
**Poor Adherence**	−1.2% ± 1.6% (n = 26)		−2.0% ± 1.3% (n = 25)	
**Improvement in Adherence**	−2.8% ± 2.1% (n = 26)	0.002	−2.9% ± 1.7% (n = 26)	0.142
**No Improvement in Adherence**	−1.3% ± 1.6% (n = 57)		−2.2% ± 1.9% (n = 57)	

^1^ Wilcoxon Rank Sum test.

**Table 4:** Correlation between mean change in total patient satisfaction and mean change in HbA1c over time (n = 83), mean ± SD.

	Δ from Baseline to 3 Months	Correlation Coefficient	*p*-Value ^1^	Δ from Baseline to 6 Months	Correlation Coefficient	*p*-Value ^1^
**Total Satisfaction**	+3.4 ± 11.8	–0.289	0.008	+4.4 ± 15.4	−0.062	0.578
**HbA1c**	−1.8% ± 1.9%			−2.4% ± 1.9%		

^1 ^ Spearman Rank Order Correlation.

## Data Availability

The data presented in this study are available upon request from the corresponding author. The data are not publicly available due to privacy restrictions.
